# Special Issue “Ion Pumps: Molecular Mechanisms, Structure, Physiology”

**DOI:** 10.3390/ijms27041696

**Published:** 2026-02-10

**Authors:** Sergey A. Siletsky

**Affiliations:** 1 Belozersky Institute of Physical-Chemical Biology, Lomonosov Moscow State University, 119991 Moscow, Russia; siletsky@belozersky.msu.ru; 2 Faculty of Bioengeneering and Bioinformatics, Lomonosov Moscow State University, 119991 Moscow, Russia

This Special Issue, entitled “Ion Pumps: Molecular Mechanisms, Structure, and Physiology,” is devoted to studies of membrane proteins that transport ions. These proteins play a key role in biological membranes and are widespread in all groups of living organisms. Among them, ion pumps play a special role, directly converting energy from various sources, including ATP, sunlight, and redox reactions, into potential energy by pumping ions up their electrochemical concentration gradients. This Special Issue includes a total of seven articles (Contributions), which cover a wide range of fundamental and applied topics and present new information on ion pumps and ion-transporting membrane proteins. These topics include the following: elucidation of the structure and functional properties of ion pumps and related enzymes, elucidation of the mechanisms of their inhibition and activation, the role of these proteins in intracellular and intercellular signaling, new methodological approaches to their study, and their biomedical and biotechnological significance.

Deciphering the molecular mechanisms of ion membrane pumps remains one of the most important fundamental tasks of biophysics and bioenergetics. Contribution 1 of this Special Issue is devoted to a theoretical study of the molecular mechanism of a member of heme-copper oxidase superfamily, cytochrome oxidase (COX) from *Rhodobacter sphaeroides*, using computer modeling methods. Heme-copper terminal oxidases, a superfamily that includes prokaryotic oxidases and mitochondrial cytochrome oxidase, are membrane enzymes that utilize the energy of high-energy electron transfer from cytochromes c or quinols to molecular oxygen to generate proton-motive force. During the COX catalytic cycle, four electrons are sequentially transferred from the P-side of the membrane to the hydrophobic core of the protein via the Cu_A_ and heme *a* redox-centers to the catalytic binuclear center (BNC), consisting of the high-spin heme α_3_ iron and the copper ion Cu_B_. Four protons, necessary for the conversion of O_2_ into two water molecules, are transferred from the opposite side of the membrane to BNC. The movement of protons and electrons from opposite sides of the membrane generates a proton-motive force via the redox loop mechanism [1]. The other half of the free energy is conserved due to the transmembrane electrogenic transfer of four more protons during the catalytic cycle, i.e., via the “proton pump” mechanism [2].

The sequential transfer of two electrons to oxidized Cu_B_ and heme *a*_3_, denoted by the O_H_ → E_H_ and E_H_ → R transitions between the intermediate states of BNC, constitutes the reductive half-reaction of the catalytic cycle. It is immediately followed by the binding of an oxygen molecule to heme *a*_3_ and an oxidative half-reaction (P → F and F → O_H_ transitions), in which two more electrons are transferred to BNC. According to the available data, during each of the single-electron transitions of COX, the transfer of the substrate (for water formation) proton to BNC and the pumped proton through the membrane occurs [3,4,5,6,7,8]. Two proton conduction pathways (D- and K-channels) are involved in the proton transfer from the N-side of the membrane. The K-channel conducts substrate protons in the reductive half-reaction, whereas all pumped protons and substrate protons in the oxidative half-reaction are transferred through the D-channel [9,10,11,12].

Clearly, the transport of pumped and substrate protons through the D and K channels must be tightly regulated. This is particularly important for the O_H_ → E_H_ transition, which involves both proton channels and was chosen by the authors of Contribution 1 for theoretical study. In the O_H_ → E_H_ transition [13], electron density is first partially shifted to heme *a*_3_, with simultaneous transfer of the pumped proton through channel D to the proton loading site (PLS) above the BNC. Then, the electron, shared between hemes *a* and *a*_3_, is transferred to Cu_B_, simultaneously with the translocation of the substrate proton to the BNC via channel K. Finally, the pumped proton from the PLS is released to the P side of the membrane.

How is proton transport through the channels controlled? How is substrate proton transfer through the K channel ensured only after the pumped proton has been transferred through channel D? The authors of Contribution 1 attempt to advance the search for answers to these questions. The central question addressed by the authors is a theoretical study of the mutual influence of proton-conducting pathways on each other in states of varying protonation of their key residues. The authors used a model of the E_H_ state of the catalytic cycle, in which the electron is located on Cu_B_, and the substrate proton has not yet reached the catalytic center. Using theoretical approaches, they modeled various protonation states of the D and K channels, analyzed the mutual influence of amino acid residues on each other and on the degree of hydration within each channel, conducted a conformational analysis of the protein side chain residues, calculated electrostatic interactions between different pairs of residues, and so on.

For the model under study, it was established that each channel has its own independent regulation of proton conductance. The authors concluded that the regulation of proton transfer through two channels in the correct sequence during the one-electron transition from O_H_ to E_H_ is likely mediated by factors such as the appearance of the pumped proton in the PLS and/or changes in the redox states of the redox centers within the BNC. They also rightly note that the model they used cannot yet be considered fully representative of all events within the transition from O_H_ to E_H_ of COX, particularly those that must occur after proton transfer to PLS and before proton transfer through the K channel. Nevertheless, an important step has been taken in this direction, providing valuable information about this transition in the cytochrome oxidase catalytic cycle, as well as about the mechanism of control and regulation of proton transfer in D and K channels in general.

The development of new specific methods for real-time measurement of the activity of membrane ion-transport proteins is of great importance both for their structural and functional studies and for many related biomedical and biotechnological applications. Contribution 2 focuses on a membrane protein that also utilizes redox energy to generate proton-motive force. The authors developed, tested, and validated a new assay for measuring the activity of membrane nitrate reductase (Nar), the first player in the denitrification process in the dissimilatory anaerobic electron transport chain in bacteria. Functionally, Nar remains poorly understood, despite the elucidation of its three-dimensional structure [14]. During its catalytic cycle, Nar is thought to oxidize ubiquinol near the P-side of the membrane, releasing two protons into the periplasm. Two electrons from ubiquinol are transferred via two hemes to a nitrate-reducing catalytic center near the N-side of the membrane, presumably resulting in the generation of proton-motive force via a redox loop mechanism. The catalytic center of Nar, where nitrate is reduced to nitrite, contains a Mo atom coordinated by the sulfur atoms of two molecules of the specific MGD cofactor.

The new method developed by the authors offers several clear advantages, including specificity and reliability for determining endogenous Nar activity in real time, the use of the native lipid environment of Nar, and the use of endogenous natural substrates and complex I as the sole coupling enzyme. A key requirement was the need to establish conditions under which the Nar catalytic cycle is the rate-limiting step in the fully coupled electron transfer reaction from complex I to Nar. The authors were able to develop such conditions and demonstrate that Nar is the rate-limiting enzyme in the coupled reaction of complex I/Nar, thereby fully confirming the validity of this activity measurement method.

It is particularly important to note that membrane nitrate reductase (Nar) is an enzyme essential for the virulence of a number of enteropathogenic bacteria colonizing the intestine under near-anaerobic conditions. Deletions of Nar and other nitrate reductases in pathogenic *Escherichia coli* or *Salmonella enterica* result in a loss of virulence in these bacterial pathogens [15,16]. The proposed new method may prove useful in the future search for new antimicrobial drugs targeting Nar. In addition to its biomedical value, the new method has broad potential for application in biotechnology, ecology, and environmental protection. For example, it can be used to remove nitrates in water purification and in the food industry.

In addition to investigating the molecular mechanisms by which membrane transport proteins perform their transport functions, other aspects and areas of their study have recently acquired significant fundamental and applied significance. This Special Issue presents these areas, including the key role of membrane transport pumps in intracellular and intercellular signaling pathways (Contributions 3 and 5); the regulation of membrane transport protein activity, their interactions with the lipid microenvironment, and the incorporation of membrane transport proteins into cell membranes (Contributions 4 and 7); the biomedical role of membrane transporters in maintaining cellular acid/base and ion and tissues homeostasis, as well as their significance in the development and progression of a wide range of pathological disorders and diseases (Contributions 5 and 6).

Recent research has shown a trend toward discovering and studying new functions of proteins whose “primary” functions have been studied for a relatively long time. Contribution 3 reviews the wide range of studies of Na^+^/K^+^-ATPase (NKA), a protein with multifaceted functions involved in many important cellular processes. NKA belongs to the P-type ATPase superfamily (P2-type ATPase family). This superfamily is a large, ancient one of primary active pumps with diverse substrate specificities, from H^+^ to phospholipids [17]. Unlike V- and F-type ATPases, which perform rotational catalysis, P-type ATPases form a covalently phosphorylated intermediate and undergo significant conformational changes during the catalytic cycle. 

The main fundamental function of Na^+^/K^+^-ATPase, which is ubiquitously present in animal cells, is to pump three sodium and two potassium ions using the energy of ATP hydrolysis [18,19]. This transport creates and maintains electrochemical transmembrane gradients of these ions, which are necessary for various cellular processes, including electrical excitability, secondary transport, maintenance of osmotic balance, and others. On the other hand, in recent years, an increasing number of studies have demonstrated that endogenous cardiac glycosides (CGs) synthesized in the body can complexly affect NKA activity [20,21,22]. NKA acts as a receptor for these compounds in a variety of cell types, triggering a variety of signaling pathways and causing significant morphological and physiological effects. Over the past two decades, this NKA signaling function has evolved into an incredibly extensive, previously unimagined signaling network that is involved in many aspects of animal cell life [20].

Contribution 3 summarizes the results of studies published in recent years regarding the interaction of NKA and CGs. Among other things, it discusses the emerging functions of each NKA subunit (α, β, and γ (or FXDY)), in particular the additional functions of the β subunits in cell adhesion and motility, and recent data linking the expression of various FXYD family members to various cancer types and the potential prognostic role of FXYD in cancer recurrence. The review also discusses the multiplicity and tissue specificity of NKA subunit isoforms, the diversity of exogenous and endogenous CGs, and the signaling pathways triggered by CG binding to NKA.

According to current concepts, the lipid environment of membrane proteins can influence their activity both through general lipid–protein interactions and through direct chemical interactions of lipid molecules with specific lipid-binding sites in the protein. Contribution 4 is also devoted to the regulation of the P-type ATPase superfamily, but from a different family, P3. These are plasma membrane H^+^-ATPases found in plants, fungi, and protozoa [23]. The catalytic cycle of P-type ATPases is characterized by significant conformational rearrangements, which makes these proteins a convenient model for studying the influence of the lipid environment on the structural rearrangements key to their activity. Specific lipid–protein interactions of P2-type ATPases were described in detail previously [24]. The authors of Contribution 4 extend their study of lipid–protein interactions to members of the P3 family, using isoform 2 of H^+^-ATPase (AHA2) from the model plant *Arabidopsis thaliana* as an example.

The previously described mechanism of AHA2 activation is mediated by the effects of autoinhibitory terminal regulatory R-domains, the binding of which by regulatory proteins leads to the displacement of the R-domain from the active site of the enzyme [25,26]. However, it turns out that another mechanism for regulating the activity of this H^+^-ATPase in plants is possible, which can be explained by specific interactions with membrane lipid molecules. The authors of Contribution 4 used a C-terminally truncated version of AHA2 lacking the regulatory R-domain. This allowed them to insert the purified enzyme in a constitutively active state into proteoliposomes with a defined phospholipid composition and investigate the functionally direct influence of the lipid environment on AHA2 activity. As it turned out, AHA2 lacking the R domain was specifically stimulated by anionic phospholipids. Using molecular dynamics, the authors identified the AHA2 sites that specifically interact with anionic phospholipids and assessed their conservation among the P3 ATPase family and the entire P superfamily. The authors also proposed possible explanations for the effects of AHA2 activation caused by the binding of lipid molecules to these sites. Further studies of these sites using site-directed mutagenesis are planned. The obtained results and conclusions are of fundamental importance for elucidating the mechanisms of regulation and evolution of P-type ATPases.

Osteoporosis, a systemic, chronic, progressive skeletal disease leading to an increased risk of fractures, is increasingly gaining importance globally as a socially significant condition and an increasingly serious problem due to demographic changes in many countries favoring an older population. Osteoporosis results from an imbalance between bone resorption and formation and is widespread among people over the age of 50 (20–30% of the population). On the other hand, astronauts experience a significant loss of bone mass (1–2% per month) in zero-gravity conditions, counteracting which is one of the most important challenges in space exploration. While most osteoporosis research focuses on the individual effects of environmental or genetic factors on bone tissue, the authors of Contribution 5 of the Special Issue propose a new approach to studying the synergistic effects of these factors.

The authors of Contribution 5 were the first to identify the *ATP6V1H* gene in 2016 as playing a key role in bone homeostasis and as a causative gene for osteoporosis. *ATP6V1H* is the gene encoding the H-subunit of V-ATPase, which connects the V_1_ and V_O_ domains. Unlike P-type ATPases, V-ATPases utilize a fundamentally different mechanism for pumping protons across membranes—a rotational motion generated by ATP hydrolysis. Originally discovered in plant and yeast vacuoles, V-ATPases are ubiquitous in mammals in the plasma membrane of specialized cells such as osteoclasts, which play a key role in bone resorption. In Contribution 5, the authors used *ATP6V1H*-deficient mice and compared their bone tissue characteristics with wild-type mice using a wide range of biophysical and biochemical approaches. The originality of their approach lies in the combination of a genetic factor (*ATP6V1H* deficiency) and an environmental one by simulating microgravity exposure using tail suspension (the hindlimb suspension (HLS) model in rodents) [27]. The HLS model mimics many of the physiological changes associated with spaceflight, as well as prolonged bed rest.

The authors of Contribution 5 demonstrated non-trivial links between signaling pathways (including the transcription factors Fos and Jun, Scr kinase, and integrin receptors), the effects of *ATP6V1H* gene deficiency, and microgravity in the development of osteoporosis. Data were obtained directly indicating the interaction of *ATP6V1H* with subunits of integrin proteins, receptors responsible for interaction with the extracellular matrix and the transmission of intercellular signals. Through interaction with these subunits, *ATP6V1H* is thought to modulate osteoclast function and bone resorption. The authors’ data highlight complex mechanisms of interaction between various factors in the development of osteoporosis and the important role of Src in mediating the combined effects of microgravity and *ATP6V1H* gene knockout. These results expand our understanding of the mechanisms of bone loss in microgravity and pave the way for the development of potential *ATP6V1H*-based bone loss treatments and new osteoporosis drugs.

Maintaining acid-base homeostasis in the body is fundamental. The solute carrier 4 (SLC4) family is a major group of bicarbonate transporters that play a key role in pH regulation and transepithelial electrolyte movement [28,29]. Contribution 6 examines the role of secondary transmembrane transporters of the solute carrier 4 (SLC4) family in human disease. In mammals, the SLC4 family consists of 10 genes. SLC4 family HCO_3_^−^ transporters (including electroneutral Cl^−^/HCO_3_^−^ anion exchangers, electrogenic and electroneutral Na^+^-dependent cotransporters, etc.) are widely expressed throughout the body, playing a fundamental role in regulating intracellular and extracellular pH, as well as in the transepithelial secretion and absorption of bicarbonate ions and other electrolytes in various tissues. They are involved in maintaining the excretory and digestive systems, neuronal and cardiovascular efficiency, promoting the development and homeostasis of tooth enamel and bone, regulating the reproductive system, and much more.

The review of SLC4 family transporters (Contribution 6) comprehensively summarizes the latest data on diseases caused by mutations in SLC4 family genes, presents current treatment strategies, and describes the clinical presentation of these diseases. The review discusses the potential biomedical significance of these transport proteins and their future potential as new therapeutic and diagnostic targets. Current approaches in terranostics medicine involve the development of new drugs with both diagnostic and therapeutic properties. Such molecules will be able to specifically target and neutralize (or cure) pathological cells in the body. This review, which systematizes existing data on the role of this extremely important family of membrane transport proteins, may be very useful for the development of new methods and drugs in the field of terranostics.

One of the most important problems in the study of membrane ion pumps is the elucidation of the intracellular mechanisms of their maturation and specific incorporation (ensuring unidirectional orientation) into cell membranes. Contribution 7 is devoted to the study of the mechanisms of unidirectional incorporation of light-dependent microbial rhodopsin, a membrane proton pump from Exiguobacterium sibiricum (ESR), into biological membranes. 

Microbial rhodopsins use retinal as the main light pigment, harnessing light energy for ion transport or sensory functions [30]. Thanks to metagenomic studies, many new retinal proteins have recently been discovered [31]. A unique feature of ESR is the presence of a lysine residue instead of the key aspartate or glutamate residues that typically function as the primary proton donor for the Schiff base in other retinal pumps [32,33]. Accordingly, the electrogenic mechanism of proton pumping in ESR also has its own unique characteristics [34]. 

Incorporation of membrane protein pumps into proteoliposomes (lipid vesicles that mimic biomembranes) provides an opportunity to model the mechanisms of their targeted incorporation into natural lipid environments and study the transport properties of such proteins. Conventional procedures for incorporating purified proteins into proteoliposomes can sometimes result in a predominantly directional protein orientation, but cannot lead to the completely unidirectional protein orientation observed in cellular membranes. The addition of artificial soluble modules to the N- and C-termini of target membrane proteins can serve as a tool for increasing the efficiency of their unidirectional orientation in proteoliposomes [35], mimicking the natural mechanisms used by cells to incorporate membrane proteins into cell membranes. 

The authors of Contribution 7 obtained ESR fusions with three soluble protein partners (McCherry fluorescent protein or thioredoxin at the C-terminus and the chaperone Caf1M at the N-terminus). Comparative functional studies of the resulting fusion proteins, their ability to incorporate into proteoliposomes, and a time-resolved study of the mechanisms of the electrogenic proton pump demonstrated the effects of terminal modules on ESR orientation in proteoliposomes, identified the conditions for efficient unidirectional incorporation of the hybrid ESR into proteoliposomes, and, consequently, for high transport activity of this proton pump. In conclusion, the authors’ approaches and results may be useful for the targeted incorporation of other membrane proteins into artificial membranes, expanding their functional studies and biotechnological applications.

## References

[1] Mitchell P. (1966). Chemiosmotic coupling in oxidative and photosynthetic phosphorylation. Biol. Rev. Camb. Philos. Soc..

[2] Wikstrom M. (1977). Proton pump coupled to cytochrome *c* oxidase in mitochondria. Nature.

[3] Verkhovsky M.I., Jasaitis A., Verkhovskaya M.L., Morgan J.E., Wikstrom M. (1999). Proton translocation by cytochrome *c* oxidase. Nature.

[4] Siletsky S., Kaulen A.D., Konstantinov A.A. (1999). Resolution of electrogenic steps coupled to conversion of cytochrome *c* oxidase from the peroxy to the ferryl-oxo state. Biochemistry.

[5] Siletsky S.A., Pawate A.S., Weiss K., Gennis R.B., Konstantinov A.A. (2004). Transmembrane charge separation during the ferryl-oxo → oxidized transition in a non-pumping mutant of cytochrome *c* oxidase. J. Biol. Chem..

[6] Bloch D., Belevich I., Jasaitis A., Ribacka C., Puustinen A., Verkhovsky M.I., Wikstrom M. (2004). The catalytic cycle of cytochrome *c* oxidase is not the sum of its two halves. Proc. Natl. Acad. Sci. USA.

[7] Ruitenberg M., Kannt A., Bamberg E., Fendler K., Michel H. (2002). Reduction of cytochrome c oxidase by a second electron leads to proton translocation. Nature.

[8] Lepp H., Salomonsson L., Zhu J.-P., Gennis R.B., Brzezinski P. (2008). Impaired proton pumping in cytochrome c oxidase upon structural alteration of the D pathway. Biochim. Biophys. Acta.

[9] Konstantinov A.A., Siletsky S., Mitchell D., Kaulen A., Gennis R.B. (1997). The roles of the two proton input channels in cytochrome *c* oxidase from *Rhodobacter sphaeroides* probed by the effects of site-directed mutations on time resolved electrogenic intraprotein proton transfer. Proc. Natl. Acad. Sci. USA.

[10] Vilhjalmsdottir J., Albertsson I., Blomberg M.R.A., Adelroth P., Brzezinski P. (2020). Proton transfer in uncoupled variants of cytochrome c oxidase. FEBS Lett..

[11] Belevich I., Gorbikova E., Belevich N.P., Rauhamaki V., Wikstrom M., Verkhovsky M.I. (2010). Initiation of the proton pump of cytochrome c oxidase. Proc. Natl. Acad. Sci. USA.

[12] Siletsky S.A., Konstantinov A.A. (2012). Cytochrome c oxidase: Charge translocation coupled to single-electron partial steps of the catalytic cycle. Biochim. Biophys. Acta.

[13] Belevich I., Bloch D.A., Belevich N., Wikstrom M., Verkhovsky M.I. (2007). Exploring the proton pump mechanism of cytochrome *c* oxidase in real time. Proc. Natl. Acad. Sci. USA.

[14] Bertero M.G., Rothery R.A., Palak M., Hou C., Lim D., Blasco F., Weiner J.H., Strynadka N.C. (2003). Insights into the respiratory electron transfer pathway from the structure of nitrate reductase A. Nat. Struct. Biol..

[15] Spees A.M., Wangdi T., Lopez C.A., Kingsbury D.D., Xavier M.N., Winter S.E., Tsolis R.M., Baumler A.J. (2013). Streptomycin-induced inflammation enhances *Escherichia coli* gut colonization through nitrate respiration. mBio.

[16] Lopez C.A., Rivera-Chavez F., Byndloss M.X., Baumler A.J. (2015). The Periplasmic Nitrate Reductase NapABC Supports Luminal Growth of *Salmonella enterica* Serovar Typhimurium during Colitis. Infect. Immun..

[17] Palmgren M. (2023). P-type ATPases: Many more enigmas left to solve. J. Biol. Chem..

[18] Skou J.C. (1957). The influence of some cations on an adenosine triphosphatase from peripheral nerves. Biochim. Biophys. Acta.

[19] Morth J.P., Pedersen B.P., Toustrup-Jensen M.S., Sorensen T.L., Petersen J., Andersen J.P., Vilsen B., Nissen P. (2007). Crystal structure of the sodium-potassium pump. Nature.

[20] Pratt R.D., Brickman C.R., Cottrill C.L., Shapiro J.I., Liu J. (2018). The Na/K-ATPase Signaling: From Specific Ligands to General Reactive Oxygen Species. Int. J. Mol. Sci..

[21] Lopina O.D., Fedorov D.A., Sidorenko S.V., Bukach O.V., Klimanova E.A. (2022). Sodium Ions as Regulators of Transcription in Mammalian Cells. Biochemistry.

[22] Obradovic M., Sudar-Milovanovic E., Gluvic Z., Banjac K., Rizzo M., Isenovic E.R. (2023). The Na^+^/K^+^-ATPase: A potential therapeutic target in cardiometabolic diseases. Front. Endocrinol..

[23] Palmgren M.G., Nissen P. (2011). P-type ATPases. Annu. Rev. Biophys..

[24] Hossain K.R., Clarke R.J. (2019). General and specific interactions of the phospholipid bilayer with P-type ATPases. Biophys. Rev..

[25] Axelsen K.B., Venema K., Jahn T., Baunsgaard L., Palmgren M.G. (1999). Molecular dissection of the C-terminal regulatory domain of the plant plasma membrane H^+^-ATPase AHA2: Mapping of residues that when altered give rise to an activated enzyme. Biochemistry.

[26] Fuglsang A.T., Visconti S., Drumm K., Jahn T., Stensballe A., Mattei B., Jensen O.N., Aducci P., Palmgren M.G. (1999). Binding of 14-3-3 protein to the plasma membrane H^+^-ATPase AHA2 involves the three C-terminal residues Tyr(946)-Thr-Val and requires phosphorylation of Thr(947). J. Biol. Chem..

[27] Morey-Holton E.R., Globus R.K. (1998). Hindlimb unloading of growing rats: A model for predicting skeletal changes during space flight. Bone.

[28] Parker M.D., Boron W.F. (2013). The divergence, actions, roles, and relatives of sodium-coupled bicarbonate transporters. Physiol. Rev..

[29] Liu Y., Yang J., Chen L.M. (2015). Structure and Function of SLC4 Family [Formula: See text] Transporters. Front. Physiol..

[30] Ernst O.P., Lodowski D.T., Elstner M., Hegemann P., Brown L.S., Kandori H. (2014). Microbial and animal rhodopsins: Structures, functions, and molecular mechanisms. Chem. Rev..

[31] Pushkarev A., Beja O. (2016). Functional metagenomic screen reveals new and diverse microbial rhodopsins. ISME J..

[32] Balashov S.P., Petrovskaya L.E., Imasheva E.S., Lukashev E.P., Dioumaev A.K., Wang J.M., Sychev S.V., Dolgikh D.A., Rubin A.B., Kirpichnikov M.P. (2013). Breaking the carboxyl rule: Lysine 96 facilitates reprotonation of the Schiff base in the photocycle of a retinal protein from *Exiguobacterium sibiricum*. J. Biol. Chem..

[33] Gushchin I., Chervakov P., Kuzmichev P., Popov A.N., Round E., Borshchevskiy V., Ishchenko A., Petrovskaya L., Chupin V., Dolgikh D.A. (2013). Structural insights into the proton pumping by unusual proteorhodopsin from nonmarine bacteria. Proc. Natl. Acad. Sci. USA.

[34] Siletsky S.A., Mamedov M.D., Lukashev E.P., Balashov S.P., Dolgikh D.A., Rubin A.B., Kirpichnikov M.P., Petrovskaya L.E. (2016). Electrogenic steps of light-driven proton transport in ESR, a retinal protein from *Exiguobacterium sibiricum*. Biochim. Biophys. Acta.

[35] Ritzmann N., Thoma J., Hirschi S., Kalbermatter D., Fotiadis D., Muller D.J. (2017). Fusion Domains Guide the Oriented Insertion of Light-Driven Proton Pumps into Liposomes. Biophys. J..

